# Incidence and management of patients with colorectal cancer and synchronous and metachronous colorectal metastases: a population‐based study

**DOI:** 10.1002/bjs5.50299

**Published:** 2020-06-16

**Authors:** V. Väyrynen, E.‐V. Wirta, T. Seppälä, E. Sihvo, J.‐P. Mecklin, K. Vasala, I. Kellokumpu

**Affiliations:** ^1^ Departments of Gastrointestinal Surgery Jyväskylä Finland; ^2^ Thoracic Surgery Jyväskylä Finland; ^3^ Oncology Central Hospital of Central Finland Jyväskylä Finland; ^4^ Faculty of Sports and Health Sciences University of Jyväskylä Jyväskylä Finland; ^5^ Department of Gastrointestinal Surgery Tampere University Hospital Tampere Finland; ^6^ Department of Gastrointestinal Surgery Helsinki University Hospital Helsinki Finland; ^7^ Department of Surgical Oncology Johns Hopkins Hospital Baltimore Maryland USA

## Abstract

**Background:**

This population‐based study aimed to examine the incidence, patterns and results of multimodal management of metastatic colorectal cancer.

**Methods:**

A retrospective population‐based study was conducted on patients with metastatic colorectal cancer in Central Finland in 2000–2015. Clinical and histopathological data were retrieved and descriptive analysis was conducted to determine the pattern of metastatic disease, defined as synchronous, early metachronous (within 12 months of diagnosis of primary disease) and late metachronous (more than 12 months after diagnosis). Subgroups were compared for resection and overall survival (OS) rates.

**Results:**

Of 1671 patients, 296 (17·7 per cent) had synchronous metastases, and 255 (19·6 per cent) of 1302 patients with resected stage I–III tumours developed metachronous metastases (94 early and 161 late metastases). Liver, pulmonary and intraperitoneal metastases were the most common sites. The commonest metastatic patterns were a combination of liver and lung metastases. The overall metastasectomy rate for patients with synchronous metastases was 16·2 per cent; in this subgroup, 3‐ and 5‐year OS rates after any resection were 63 and 44 per cent respectively, compared with 7·1 and 3·3 per cent following no resection (*P* < 0·001). The resection rate was higher for late than for early metachronous disease (28·0 *versus* 17 per cent respectively; *P* = 0·048). Three‐ and 5‐year OS rates after any resection of metachronous metastases were 78 and 62 per cent respectively *versus* 42·1 and 18·2 per cent with no metastasectomy (*P* < 0·001). Similarly, 3‐ and 5‐year OS rates after any metastasectomy for early metachronous metastases were 57 and 50 per cent *versus* 84 and 66 per cent for late metachronous metastases (*P* = 0·293).

**Conclusion:**

The proportion of patients with metastatic colorectal cancer was consistent with that in earlier population‐based studies, as were resection rates for liver and lung metastases and survival after resection. Differentiation between synchronous, early and late metachronous metastases can improve assessment of resectability and survival.

## Graphical abstract

In a study of the incidence and management of metastatic colorectal cancer in a population‐based cohort, 17·7 per cent of 1671 patients with colorectal cancer had synchronous metastases, and 19·6 per cent of 1302 patients who had resection of stage I–III tumours developed metachronous metastases. In the whole population, the proportion of patients with any metastasis was 33·0 per cent.

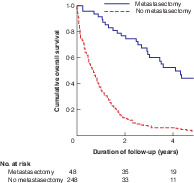

Metastasis from colorectal cancer

## Abstract (es)

**Antecedentes:**

El objetivo de este estudio de base poblacional fue analizar la incidencia, la forma de presentación y los resultados del tratamiento multimodal del cáncer colorrectal metastásico (*metastatic colorectal ca*ncer, mCRC).

**Métodos:**

Se realizó un estudio retrospectivo de base poblacional en pacientes con mCRC en la región central de Finlandia entre 2000 a 2015. Se recuperaron los datos clínicos e histopatológicos y se realizó un análisis descriptivo con el objetivo de analizar la forma de presentación de la enfermedad metastásica. La enfermedad metastásica se definió como sincrónica, metacrónica precoz (< 12 meses) y metacrónica tardía (> 12 meses después del diagnóstico de la enfermedad primaria) y se compararon las tasas de resección y de supervivencia global (*overall survival*, OS) en estos subgrupos.

**Resultados:**

De los 1.671 pacientes revisados, 296 (17,7%) presentaron metástasis sincrónicas, mientras que de los 1.302 pacientes resecados en estadios I‐III, 255 (19,6%) tuvieron metástasis metacrónicas: 94 precoces y 161 tardías. La localización metastásica más frecuente fue el hígado, los pulmones y el peritoneo. La combinación más frecuente fue la de metástasis hepáticas y pulmonares. La tasa de resección para pacientes con metástasis sincrónicas fue del 16,2%; en este subgrupo, la OS a 3 y 5 años después de cualquier tipo de resección fue del 62,6% y 44,2% *versus* 7,1% y 3,3% en los pacientes sin resección, respectivamente (*P* < 0,001). La tasa de resección fue mayor en la enfermedad metacrónica tardía que en la enfermedad metacrónica precoz (28% *versus* 17%, *P* = 0,048). Las tasas de OS a 3 y 5 años después de cualquier resección en los casos de metástasis metacrónicas fueron del 77,8% y 61,9% *versus* 42,1% y 18,2% en los pacientes sin metastasectomía, *P* < 0,001. Las tasas de OS a 3 y 5 años después de cualquier metastasectomía en los casos de metástasis metacrónicas precoces fueron del 57,4% y 50,3%, *versus* 84,3% y 65,6% en las tardías (*P* = 0,29)

**Conclusión:**

La proporción de pacientes con mCRC fue similar a la de estudios anteriores de base poblacional, así como las tasas de resección para metástasis hepáticas y pulmonares y la supervivencia después de la resección. Diferenciar entre metástasis sincrónicas, metacrónicas precoces y tardías puede mejorar la posibilidad de resecabilidad y la supervivencia.

## Introduction

Colorectal carcinoma is the third most common cancer worldwide^1^, ^2^ and the fourth leading cause of cancer death^1^. Over the years, many improvements have been made in the management of primary and metastatic colorectal cancer^3^, ^4^, ^5^. These include diagnostic procedures, extended indications for resection of metastatic disease, improvement in perioperative care, and the development of effective neoadjuvant, adjuvant and palliative treatments. The impact of these improvements at a population level is not well known^6^.

Only a few population‐based studies^7^, ^8^, ^9^ have been published assessing the incidence and patterns of metastatic colorectal cancer and survival. According to these studies, 15–30 per cent of patients with colorectal cancer have synchronous or metachronous metastases. The observed rate of synchronous liver metastasis ranges from 14·5 to 19 per cent^7^, ^10^, ^11^, ^12^, ^13^, and that of metachronous liver metastases ranges from 8·1 to 12·8 per cent^8^, ^10^, ^11^, ^13^.

The aim of this study was to examine the incidence and patterns of metastatic disease, surgical management and survival in patients with metastatic colorectal cancer by reviewing all diagnosed colorectal cancers from 2000 to 2015 in a well defined population in Central Finland.

## Methods

According to Finnish healthcare policy, all municipalities are responsible for arranging specialized hospital care for their residents. Each hospital district organizes and provides specialized hospital care for the population in its area. The Central Hospital of Central Finland is the only gastroenterological surgery unit in the Central Finland hospital district. The annual population of the area was obtained from Statistics Finland, and averaged around 270 000 during the study period, from 1 January 2000 to 31 December 2015. All patients with primary and metastatic colorectal cancer are managed in this hospital, with no referrals to other hospitals.

Patients diagnosed with primary colorectal cancer during the study interval were identified using the histopathological registry of the hospital, which covers all colorectal cancers diagnosed in the area. Clinical and histopathological data, as well as recurrence data, were retrieved retrospectively from hospital records. Colonoscopy, thoracoabdominal CT, endorectal ultrasonography and pelvic MRI were used to diagnose and stage primary colorectal tumours. All patients with colorectal primary and metastatic disease were discussed in multidisciplinary team (MDT) meetings before definitive treatment decisions were made.

Surgery for primary colorectal cancers was performed according to international guidelines^3^, mostly with a laparoscopic approach, complete mesocolic and total mesorectal excision principles. Liver surgery was performed according to international guidelines^4^, ^5^, using intraoperative ultrasound imaging, a cavitron ultrasonic surgical aspirator and bipolar energy devices. After 2011, lung metastases were treated primarily with a thoracoscopic approach using wedge resection or segmentectomy. Tumours were staged by staff pathologists according to the UICC/TNM classification^14^.

Neoadjuvant and adjuvant treatments for primary and metastatic disease were administered according to international guidelines^3^. Since 2005, adjuvant postoperative chemotherapy for 6 months, consisting of 5‐fluorouracil (5‐FU) and oral folic acid, oral capecitabine or folic acid, 5‐FU and oxaliplatin (FOLFOX regimen), was prescribed to medically fit patients with stage III tumours or high‐risk stage II disease. Patients with liver metastases received perioperative chemotherapy with the FOLFOX regimen, with or without biologicals, according to the decision taken at the MDT meeting.

Surgery for advanced disease was performed when appropriate, according to the local MDT. Significant co‐morbidity and inadequate physical and mental performance status were contraindications for surgery. Unresectable metastatic disease was defined as the inability to achieve complete resection of all metastases, liver and lung metastases combined with more than one extrahepatic site, extensive extrahepatic metastatic disease, inability to leave at least 30–40 per cent of functional liver volume in the case of liver metastases, and progression of metastatic disease during chemotherapy.

The study was approved by the hospital administrative and ethics board (Dnro13U/2011 and 1/2016) and the National Authority for Welfare and Health (Valvira) (Dnro 3916/06.01.03.01/2016).

### Data collection

The study variables for primary tumours included age, sex, tumour location, TNM/UICC stage, and date of surgery. The date of diagnosis of metastatic disease, metastatic site, and number and size of the metastases were recorded. According to an international consensus meeting report^5^, synchronous metastases were defined as metastases detected before or at the time of diagnosis of the primary cancer. Early metachronous metastases were defined as metastases detected at or within 12 months of diagnosis of the primary tumour, and late metachronous metastases as those detected more than 12 months after diagnosis of the primary^5^.

### Outcome measures

Outcome measures included resection rate, defined as number of metastasectomies with curative intent, and overall survival (OS), defined as the percentage of patients alive at 3 and 5 years after the date of diagnosis of metastases or the start of therapy.

Follow‐up after surgery for primary tumours and metastases included carcinoembryonic antigen estimation, clinical examination, ultrasound investigation of the liver, and chest radiography every 6 months during the first 3 years, and annually thereafter up to October 2017. Further characterization of recurrent metastases was done by CT and/or MRI, and after 2005 also by CT–PET. Locally recurrent disease in the bowel was assessed by CT/pelvic MRI and endoscopy. Locoregional recurrence was defined as a recurrent tumour at the anastomotic site or locoregionally in the abdomen, and diagnosed by CT, MRI and endoscopy to ascertain whether newly diagnosed distant metastasis was absent or present. Causes of death were obtained from hospital records and the National Cause of Death Registry.

### Statistical analysis

Results are given as mean(s.d.) or median (i.q.r.) values. Pearson's χ^2^ or Fisher's exact tests were used to compare frequencies, and Student's *t* test, Mann–Whitney *U* test and Kruskal–Wallis test for continuous variables. The Kaplan–Meier method was used to calculate survival, and differences between groups were compared with the log rank test. Survival times were calculated from the date of primary surgery to the date of death or the end of follow‐up. As the number of patients with rectal cancer and a complete pathological response after chemoradiotherapy was small, these patients were included with patients with stage I disease for purposes of statistical calculation. All statistical tests were two‐sided. *P* < 0·050 was considered significant. STATA® release 11 2009 (StataCorp, College Station, Texas, USA) was used for statistical analysis.

## Results

A total of 1671 patients met the criteria of having been diagnosed and treated for colorectal cancer at Central Finland Hospital District during the study period. Baseline characteristics of the patients are shown in *Table* 1. Patients with metastatic disease were younger than those without metastases (mean 68·8 *versus* 71·1 years respectively; *P* < 0·001), with no significant differences in sex and primary tumour site distribution. Of the 1671 primary tumours 1471 (88·0 per cent) were resected. Primary tumours were resected less often in patients with metastatic colorectal cancer than in those without metastases (77·0 *versus* 93·5 per cent; *P* < 0·001).

**Table 1:** Characteristics of patients with and without distant metastases

	All patients (*n* = 1671)	No metastases (*n* = 1120)	Synchronous and metachronous metastases (*n* = 551)	*P* †
**Age (years)** *	70·3(11·3)	71·1(11·2)	68·8(11·5)	< 0·001‡
**Age group (years)**				0·005
< 65	497 (29·7)	305 (27·2)	192 (34·8)	
65–75	575 (34·4)	395 (35·3)	180 (32·7)	
> 75	599 (35·8)	420 (37·5)	179 (32·5)	
**Male sex**	904 (54·1)	606 (54·1)	298 (54·1)	0·993
**Primary tumour site**				0·871
Colon	1069 (64·0)	718 (64·1)	351 (63·7)	
Rectum	602 (36·0)	402 (35·9)	200 (36·3)	
**Side of colon**				0·472
Right	623 (58·3)	413 (57·5)	210 (59·8)	
Left	446 (41·7)	305 (42·5)	141 (40·2)	
**pT category**				< 0·001
pCR (complete response)	8 (0·5)	8 (0·7)	0 (0)	
pT1	147 (8·8)	134 (12·0)	13 (2·4)	
pT2	258 (15·4)	223 (19·9)	35 (6·4)	
pT3	832 (49·8)	584 (52·1)	248 (45·0)	
pT4	226 (13·5)	98 (8·8)	128 (23·2)	
Missing	200 (12·0)	73 (6·6)	127 (23·0)	
**pN category**				< 0·001
pN0	900 (53·9)	758 (67·7)	142 (25·8)	
pN1	342 (20·5)	202 (18·0)	140 (25·4)	
pN2	228 (13·6)	87 (7·8)	141 (25·6)	
Missing	201 (12·0)	73 (6·5)	128 (23·2)	
**M category**				< 0·001
M0	1303 (78·0)	1048 (93·6)	255 (46·3)	
M1	296 (17·7)	0 (0)	296 (53·7)	
Missing	72 (4·3)	72 (6·4)	0 (0)	
**UICC tumour stage**				< 0·001
pCR (complete response)	8 (0·5)	8 (0·7)	0 (0)	
I	342 (20·5)	308 (27·5)	34 (6·2)	
II	513 (30·7)	442 (39·5)	71 (12·9)	
III	439 (26·3)	289 (25·8)	150 (27·2)	
IV	296 (17·7)	0 (0)	296 (53·7)	
Missing	73 (4·4)	73 (6·5)	0 (0)	
**Resection of primary tumour**				< 0·001
Yes	1471 (88·0)	1047 (93·5)	424 (77·0)	
No	200 (12·0)	73 (6·5)	127 (23·0)	

Values in parentheses are percentages unless indicated otherwise;

* values are mean(s.d.).

† χ^2^ or Fisher's exact test, except

‡ Student's *t* test.

Some 200 patients (12·0 per cent) did not have surgery for the primary tumour, because of significant co‐morbidity, old age, locally unresectable primary cancer, unresectability of metastases or patient preference. Rectal carcinomas were unresectable more frequently than colonic carcinomas (15·6 *versus* 9·9 per cent respectively; *P* = 0·001). The median number of lymph nodes investigated was 12 (i.q.r. 6–17) in those who had primary tumour resection. Of patients with metastatic colorectal cancer, 51·0 per cent had regional lymph node metastases compared with 25·8 per cent of patients without metastatic disease (*Table* 1).

### Characteristics of patients with synchronous *versus* metachronous metastases

Metastatic disease was diagnosed in 551 (33·0 per cent) of the 1671 patients, of which 296 (17·7 per cent) were synchronous metastases (*Table* 2). Of 1302 resected stage I–III tumours, 255 (19·6 per cent) were metachronous, 94 (7·2 per cent) early metachronous, and 161 (12·4 per cent) late metachronous metastases. Tumour site distribution (colon *versus* rectum, or right *versus* left colon) did not influence the incidence or distribution of synchronous and metachronous liver, pulmonary or intraperitoneal metastases. The node positivity rate for primary colorectal cancers was highest in the early metachronous group (68·1 per cent) compared with the late metachronous (53·4 per cent) or synchronous (44·3 per cent) group (*P* < 0·001).

**Table 2:** Characteristics of 551 patients with synchronous and metachronous metastases

	Synchronous (*n* = 296)	Early metachronous (*n* = 94)	Late metachronous (*n* = 161)	*P* †
**Age (years)** *	68·7(11·6)	70·6(12·0)	67·8(11·0)	0·171‡
**Age group**				0·076
< 65	107 (36·1)	28 (30)	57 (35·4)	
65–75	92 (31·1)	26 (28)	62 (38·5)	
> 75	97 (32·8)	40 (43)	42 (26·1)	
**Male sex**	158 (53·4)	52 (55)	88 (54·7)	0·933
**Primary tumour site**				0·532
Colon	194 (65·5)	60 (64)	97 (60·2)	
Rectum	102 (34·5)	34 (36)	64 (39·8)	
**Side of colon**				0·488
Right	114 (58·8)	40 (67)	56 (58)	
Left	80 (41·2)	20 (33)	41 (42)	
**UICC tumour stage**				< 0·001
I	0 (0)	8 (9)	26 (16·1)	0·054§
II	0 (0)	22 (23)	49 (30·4)	
III	0 (0)	64 (68)	86 (53·4)	
IV	296 (100)	0 (0)	0 (0)	
**Most common metastatic site at presentation**				
Liver	240 (81·1)	51 (54)	83 (51·6)	< 0·001
Lung	89 (30·1)	29 (31)	66 (41·0)	0·052
Intra‐abdominal, extrahepatic	76 (25·7)	54 (57)	63 (39·1)	< 0·001
Bone	3 (1·0)	4 (4)	5 (3·1)	0·109
Brain	5 (1·7)	4 (4)	5 (3·1)	0·335
**Size of largest liver metastasis (cm)**				0·015
< 5	157 (65·4)	37 (73)	65 (78)	
≥ 5	78 (32·5)	10 (20)	17 (20)	
Missing	5 (2·1)	4 (8)	1 (1)	
**No. of liver metastases**				< 0·001
1	40 (16·7)	16 (31)	37 (45)	
2–3	62 (25·8)	15 (29)	30 (36)	
> 4	133 (55·4)	16 (31)	15 (18)	
Missing	5 (2·1)	4 (8)	1 (1)	
**Pattern of distant metastasis at diagnosis**				< 0·001
Liver only	148 (50·0)	25 (27)	52 (32·3)	
Lung only	20 (6·8)	7 (7)	34 (21·1)	
Liver and lung	52 (17·6)	8 (9)	12 (7·5)	
Liver and extrahepatic (no lung)	30 (10·1)	13 (14)	13 (8·1)	
Lung and extrahepatic (no liver)	7 (2·4)	9 (10)	14 (8·7)	
Liver, lung and extrahepatic	10 (3·4)	5 (5)	6 (3·7)	
Extrahepatic only	29 (9·8)	27 (29)	30 (18·6)	
**Resection of primary tumour**	169 (57·1)	94 (100)	161 (100)	< 0·001
**Metastasectomy**				0·007
No	248 (83·8)	78 (83)	116 (72·0)	0·048§
Yes	48 (16·2)	16 (17)	45 (28·0)	
**Liver resection**	44 of 240 (18·3)	15 of 51 (29)	31 of 83 (37)	0·003
**Lung resection**	12 of 89 (13)	6 of 29 (21)	17 of 66 (26)	0·025
**Extrahepatic, intra‐abdominal resection**	1 of 76 (1)	2 of 54 (4)	1 of 63 (2)	0·392

Values in parentheses are percentages unless indicated otherwise;

* values are mean(s.d.).

† χ^2^ or Fisher's exact test, except

‡ Student's *t* test (synchronous *versus* metachronous metastases) and

§ χ^2^ or Fisher's exact test (early *versus* late metachronous metastases).

Overall, metastatic sites and patterns, as well as the number and size of liver metastases, varied significantly between synchronous, early and late metachronous metastases (*Table* 2).

Metastatic patterns varied significantly between the study groups. The most common metastatic patterns were combinations of liver and lung metastases, followed by combined liver and extrahepatic (no lung) metastases, and combined liver, lung and extrahepatic metastases (*Table* 2). The proportion of patients having only intra‐abdominal, extrahepatic recurrences was highest in the early and late metachronous groups.

### Management and survival of patients with synchronous metastases

The primary tumour was resected in 169 (57·1 per cent) of the 296 patients with synchronous metastases. The overall metastasectomy rate was 16·2 per cent (48 of 296), and included liver resection in 36 patients (with combined radiofrequency ablation in 5), liver and lung resection in eight, and lung resections in four patients. Liver resection was performed in 44 (18·3 per cent) of the 240 patients with synchronous liver metastases, and lung resection in 12 (13 per cent) of the 89 patients with lung metastases. Of the 44 patients with synchronous liver metastases undergoing liver resection, neoadjuvant chemotherapy was given to 31 (70 per cent) and adjuvant chemotherapy to 39 (89 per cent).

**Figure 1:**
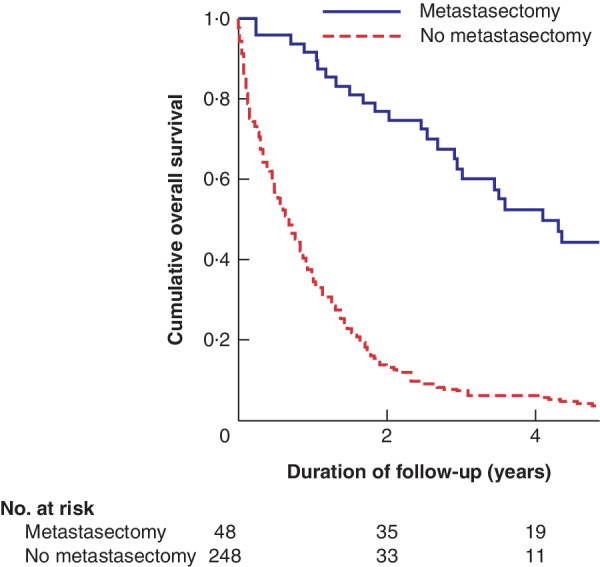
**Kaplan–Meier analysis of overall survival in 296 patients with synchronous metastases who had resection of the primary colorectal tumour with or without metastasectomy** *P* < 0·001 (log rank test).

The 3‐ and 5‐year OS rates after any resection of synchronous metastases were 63 and 44 per cent respectively compared with 7·1 and 3·3 per cent in patients who did not have resection (*P* < 0·001) (*Fig*. 1). Three‐ and 5‐year OS rates after liver resection were 64 and 44 per cent respectively *versus* 4·4 and 0·8 per cent with no liver resection (*P* < 0·001).

### Management and survival of patients with metachronous metastases

Metachronous metastases were detected in 255 (19·6 per cent) of the 1302 patients with stage I–III tumours who had bowel resection for primary colorectal cancer: early metachronous 94 (7·2 per cent) and late metachronous 161 (12·4 per cent) metastases (*Table* 2). Of these 255 patients, 61 (23·9 per cent) underwent metastasectomy. Liver resection was performed in 46 (34·3 per cent) of the 134 patients with liver metastases, and lung resection in 23 (24 per cent) of the 95 patients with lung metastases: liver only resection in 38 patients (28 per cent) and both liver and lung resection in eight (6 per cent). Of the 46 patients with metachronous liver metastases undergoing liver resection, neoadjuvant chemotherapy was given to 21 patients (46 per cent) and adjuvant chemotherapy to 41 (89 per cent). Lung‐only resections were performed in 14 (15 per cent) of the 95 patients, and combined resection of lung and intra‐abdominal extrahepatic metastases on one patient. The resection rate was higher for late than for early metachronous disease (28·0 *versus* 17 per cent respectively; *P* = 0·048).

**Figure 2:**
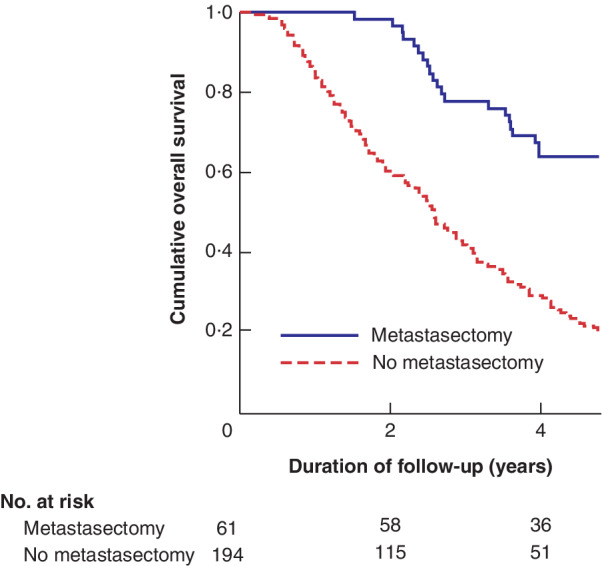
**Kaplan–Meier analysis of overall survival in 255 patients with metachronous metastases who had resection of the primary colorectal tumour with or without metastasectomy** *P* < 0·001 (log rank test).

The 3‐ and 5‐year OS rates after any resection of metachronous metastases were 78 and 62 per cent *versus* 42·1 and 18·2 per cent with no metastasectomy (*P* < 0·001) (*Fig*. 2). Three and 5‐year OS rates after any metastasectomy for early metachronous metastases were 57 and 50 per cent respectively, and for late metachronous metastases 84 and 66 per cent (*P* = 0·293). Three and 5‐year OS rates after liver resection for early metachronous metastases were 54 and 46 per cent respectively, and for late metachronous metastases 81 and 67 per cent (*P* = 0·319).

## Discussion

This population‐based study in Finland covering the years 2000–2015 showed that the proportion of patients with colorectal cancer diagnosed with synchronous (17·7 per cent) or metachronous (19·6 per cent) metastases was consistent with earlier large population‐based studies^7^, ^8^, ^9^ reporting synchronous or metachronous metastases in approximately 15–25 per cent of all patients with colorectal cancer. Overall metastasectomy, liver and lung resection rates for synchronous and metachronous metastases compared favourably with those reported in these earlier studies. In contrast to the previous study from Sweden^13^, no significant association was found between tumour sidedness and metastatic pattern.

Patients with synchronous metastatic disease had more advanced primary colorectal cancers with regard to T category and lymph node metastasis than patients without distant metastases. In general, the node positivity rate for colorectal cancer has consistently been some 40 per cent across a wide range of international studies^15^, ^16^, ^17^. In the present study, the node positivity rate was significantly higher in patients with primary colorectal cancer who developed metachronous metastases within 12 months than in patients who developed metastases 12 months or more after primary surgery. Other studies^17^, ^18^ have previously shown that high lymph node ratio is associated with the development of distant colorectal metastases and reduced disease‐free survival.

In comparison with previous population‐based studies^7^, ^8^, ^9^, ^10^, ^11^, a new division between synchronous and metachronous metastases with early and late metachronous disease was applied in the present study, resulting in a significant difference between these three groups regarding resectability of metastases and survival.

The most common metastatic sites, as in earlier studies^7^, ^8^, were liver, lungs and intra‐abdominal extrahepatic sites. Overall, the incidence, metastatic sites and patterns concurred with the values reported in other studies^7^, ^8^. The incidence of synchronous and metachronous liver metastases reported here was similar to that in other population‐based series^7^, ^8^, ^10^, ^11^, ^12^, ^13^. Liver‐only metastases accounted for 50·0 per cent of synchronous metastases and approximately one‐third of metachronous metastases. The reported incidence of synchronous and metachronous pulmonary metastases is 5–7·2 per cent^7^, ^8^, in line with the present results.

A combined metastatic pattern involving the liver was seen frequently in the synchronous and metachronous groups; combined liver and lung metastases were the most frequent, followed by combined liver and extrahepatic intra‐abdominal metastases. The rate of combined liver and lung metastases in the metachronous group was similar to that of 5–10 per cent reported in the literature^19^, ^20^, but was almost twice as high in the synchronous group.

The presence of extrahepatic metastatic disease has long been considered a contraindication for liver resection as the prognosis has been poor, especially when more than one extrahepatic site is involved. Mounting evidence currently supports resection of liver metastases and concurrent extrahepatic metastases in well selected patients^21^, ^22^. In the present study, metastasectomy focused mainly on liver and lung metastases and single extrahepatic sites.

Overall, the metastasectomy rate for synchronous (16·2 per cent) and metachronous (23·9 per cent) metastases compares favourably with those of 12–23 per cent reported in other population‐based studies^7^. Owing to the vigilant follow‐up of patients who had surgical resection for primary colorectal cancer, the metastasectomy rate in the present study was significantly higher in the metachronous group than in the synchronous group. The rate of liver resection for synchronous (18·3 per cent), early (29 per cent) and late (37 per cent) metachronous liver metastases is in line with a hepatectomy rate of 6·3–33 per cent reported in the literature^7^, ^10^, ^11^, ^13^, ^23^. Pulmonary metastasectomy is generally recommended for highly selected subsets of patients^20^. In the present series, lung resections were increasingly performed using thoracoscopic approach for synchronous (13·5 per cent), early (21 per cent) and late (26 per cent) metachronous lung metastases, and the pulmonary metastasectomy rate compares favourably with the rate of 10 per cent reported in the literature^20^.

The prognosis of patients with limited lung metastases appears to be similar to that of patients with liver metastases, with a 5‐year survival rate of 25–45 per cent after resection^24^, ^25^. Lung metastases presenting synchronously with colorectal liver metastases are considered a systemic disease, and systemic chemotherapy is the recommended initial treatment^3^. However, 5‐ and 10‐year survival rates of 45–55 and 18 per cent respectively have been achieved in highly selected patients who had liver and pulmonary resection^24^, ^25^.

The present results should be interpreted with some caution, however. The number of patients is relatively small compared with large nationwide population‐based studies. In addition, patient selection may play a significant role in the management and outcome differences between different studies. Different definitions have also been used to define synchronous and metachronous metastases, making comparison with other population‐based studies problematic. However, the population‐based design makes selection bias highly unlikely. Further strengths are reliable cancer recurrence data and detailed follow‐up of all patients using direct methods (medical chart review) and the national death registry, providing a realistic picture of the incidence and patterns of metastatic colorectal cancer, and of the potential results of therapy at a population level with modern staging, surgical techniques, oncological treatments and meticulous follow‐up.

## Disclosure

The authors declare no conflict of interest.
